# Localization of C Cycle Enzymes in Arable and Forest Phaeozems within Levels of Soil Microstructure

**DOI:** 10.3390/microorganisms11051343

**Published:** 2023-05-19

**Authors:** Anna Yudina, Olga Ovchinnikova, Vladimir Cheptsov, Dmitry Fomin

**Affiliations:** 1 Department of Soil Physics and Hydrology, V.V. Dokuchaev Soil Science Institute, Pyzhovskiy Lane, 7, Building 2, 119017 Moscow, Russia; ovchinnikova_oy@esoil.ru; 2 Department of Soil Biology, Soil Science Faculty, Lomonosov Moscow State University, Leninskie Gory, 1, 12, 119991 Moscow, Russia; cheptcov.vladimir@gmail.com; 3 Situational Analytical Center “Soil and Land Resources of Russia”, V.V. Dokuchaev Soil Science Institute, Pyzhovskiy Lane, 7, Building 2, 119017 Moscow, Russia; fomin_ds@esoil.ru; 4 Digital Twins Laboratory of Agrolandscapes (AgroDT Lab), V.V. Dokuchaev Soil Science Institute, Pyzhovskiy Lane, 7, Building 2, 119017 Moscow, Russia

**Keywords:** soil structure, soil microaggregates, ultrasonic dispersion, extracellular enzymes, microbial diversity, soil dispersion

## Abstract

Soil microbial and enzyme activities are closely related to the spatial variability of soil environmental conditions at the microscale (μm-mm). The origin and localization of the enzymes are somewhat neglected when the measured activity is used to evaluate specific soil functions. The activity of four hydrolytic enzymes (*β*-glucosidase, Cellobiohydrolase, Chitinase, Xylanase) and microbial diversity based on community-level physiological profiling were determined in samples of arable and native Phaeozems with increasing physical impact to soil solids. The level of impact on the soil solids had a significant effect on enzyme activity and depended on both the enzyme type and soil land use. The highest proportion of the activity of Xylanase and Cellobiohydrolase of arable Phaeozem was determined at the dispersion energy in the range of 450–650 J·mL^−1^ and was associated with the primary soil particles’ hierarchy level. The highest proportions of *β*-glucosidase and Chitinase activities were determined for forest Phaeozem after applying energies lower than 150 J·mL^−1^ and characterizing the level of soil microaggregates. The increased activity of Xylanase and Cellobiohydrolase in primary soil particles of arable soil compared to those in forest soil might be a reflection of the substrates being unavailable to decomposition, leading to enzyme accumulation on the solid surface. For the Phaeozems, the lower the level of soil microstructure organization, the greater the differences observed between soils of different land use type, i.e., microbial communities, associated with lower microstructure levels, were more specific to land use type.

## 1. Introduction

Soil microbial activity and subsequently enzyme activity (EA) are closely related to the spatial variability of soil environmental conditions at the microscale (μm-mm) [1]. The physical availability of organic compounds to microorganisms strongly influences the ability of microbial communities to feed and function [2,3]. Moreover, the lower the hierarchy level of soil structure organization, the more mineralized soil organic matter (SOM) is involved in its formation [4,5]. Furthermore, the higher the soil aggregation and saturation of SOM, the more protected from degradation the enzymes are [6]. It is assumed that the number of extracellular enzymes stabilized by their interaction with soil solids is much greater than the number of intracellular or extracellular enzymes associated with active microbial cells [7].

“Heterogeneity is a fundamental property of soil that is often overlooked in microbial ecology” [8]. The physical conditions of soil microhabitats, called microenvironments, which determine nutrient availability and competitive conditions, are thought to influence enzyme production by microorganisms, given the cost–benefit relationship of this process [9]. Soil microaggregates have pore sizes ranging from a few units to hundreds of µm [10]; the range of sizes also corresponds to water storage and nutrient supply [5,11], and therefore they are considered as the main habitat for soil microbiota. Smaller pores corresponding to elementary (or composite building units, [12]) and primary soil particles [5,13] contain substrate that is inaccessible to the microbiota, which is reflected in the concept of physically protected organic matter [14,15,16]. It is considered that pores 30–90 µm in size contribute primarily to the decomposition of C in soils [17]. It may seem paradoxical from an energetic perspective, but limitations in substrate availability lead to greater production of extracellular enzymes by microorganisms [18,19]. Thus, the microstructural organization of soils is closely related to the distribution of different types of organic substrates and, accordingly, the EA of soils, and vice versa.

The origin and localization of enzymes is somewhat neglected when EA is used to evaluate the specific functions of soil [20]. The allocation of enzymes according to their localization in the solid phase of soils has been called one of the most important tasks of modern enzymology [20]. “However, in this aspect, soil enzymology still badly needs new methodological approaches to the study of enzymatic activity. Until methodology has reached such perfection that these activities can be defined and investigated separately, the overall picture of soil enzymatic activity will remain incompletely understood” [21] (p. 12). Data on the dynamics of the storage and conservation of extracellular enzymes in soils depending on soil properties and, even more so, on the microstructural organization of soils is limited. Experimental data are predominantly represented by the activity of enzymes in different particle-size fractions (e.g., [22,23,24,25,26]), i.e., immobilized on the surface of the solid phase. Particle-size fractionation schemes vary considerably in terms of applied energy and used particle size limits, which are rarely justified in terms of the microstructural organization of soils. Biogeochemical models of C are rapidly seeking to incorporate metabolic and physiological parameters as well as microbial life strategies to account for microbial regulation of decomposition processes (e.g., [27,28]). To understand the mechanisms responsible for the persistence of SOM, it is necessary to understand how the organization of soil structure at the micro- and nano-scale interacts with biotic processes [29].

This paper aims to identify the redistribution of C-cycle-related enzymes and associated microbial cells within hierarchy levels of soil microstructure in two Silt Loam Phaeozems contrasting in land use type—native forest soil and arable land. Phaeozems are one of the main soil types of the Far East, Siberia, and European Russia, occupying 1.8% of the area, and formed within humid and sub-humid forest and steppe–forest zones [30].

## 2. Materials and Methods

### 2.1. Experimental Design

**Figure 1:**
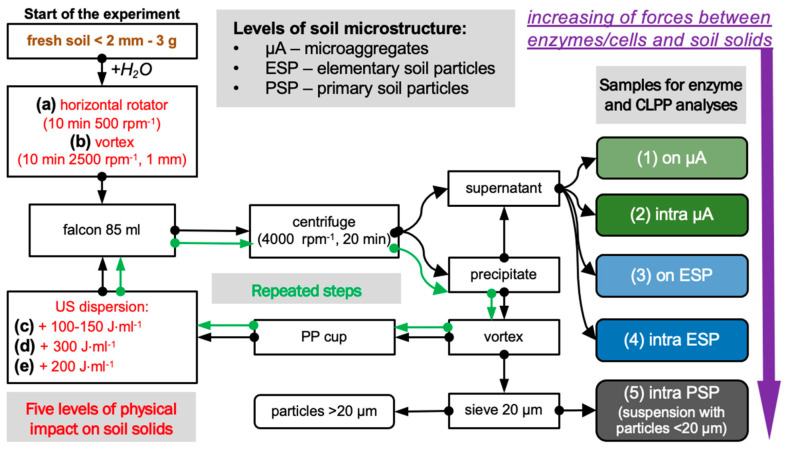
Experimental design for the isolation of enzymes and microbial cells localized on the surface and within the soil microstructural units (primary soil particles, PSP; elementary soil particles, ESP; microaggregates, µA). In total, isolation according to five levels of physical impact ((**a**–**e**), red text) on soil solids was performed from soil samples in four replicates, resulting in subsamples corresponding to surface and intra-microaggregate (on µA and intra µA), and to surface and intra-elementary soil particles (on µA and intra µA). The applied ultrasonic energy was equal to 26 J·s^−1^.

This experiment is based on a sequentially increasing physical impact on soil solids (Figure 1). According to the energy of impact, enzymes and cells were detached from the surface of the solids, and soil microstructural units of different soil hierarchy levels were dispersed [5].

In total, *five levels* of dispersion energy were used:

*1st level*: separation from the surface of microaggregates (µA) was accomplished by gently shaking the soil–water suspension (1:30 by mass) on a horizontal rotatory shaker (Multi Bio RS-24 (Biosan, Rīga, Latvia) for 10 min at 500 rpm;

*2nd level*: dispersion of µA and separation of intra-microaggregate enzymes and cells was performed by intensive mechanical shaking of the precipitate from the first step on vortex Reax-top (Heidolph Instruments, Schwabach, Germany for 10 min at a frequency of 2500 rpm and an amplitude of 1 mm for 10 min [31];

*3d level*: separation from the surface of elementary soil particles (ESP) by ultrasonic action on soil–water suspension including precipitate from the second step equal to 100–150 J·mL^−1^;

*4th level*: dispersion of ESP and separation of intra-ESP enzymes and cells was carried out using additional ultrasonic dispersion on soil solids equal to 300 J·mL^−1^; thus, the total energy of ultrasound exposure was equal to ~450 J·mL^−1^ [32,33,34];

*5th level*: separation of enzymes and cells associated with primary soil particles (PSP) was performed with excessive ultrasonic action on soil solids separated from the former step equal to 200 J·mL^−1^, and a suspension with particles less than <20 µm in size was taken for analyses. In total, the energy of soil samples was equal to 650 J·mL^−1^.

For each soil, four separate samples were used. After each step of dispersion, the soil suspension was centrifuged at 4000 rpm for 20 min, the supernatant was taken for enzyme analyses and community-level physiological profiling (CLPP), and the precipitate was subjected to the next level of dispersion energy. At the 5th level of soil dispersion, soil suspensions sieved through the mesh size equal to 20 µm were used for further analyses.

We used a horn-type ultrasonic disruptor Sonifier S-250D (Branson, MO, USA) with a ½” solid step horn with a threaded body. Calibration of the ultrasonic energy output was carried out according to a common calorimetry method (North, 1976). We used ultrasonic power equal to 26 J·mL^−1^·s^−1^ to prevent the destruction of enzymes and microbial cells.

### 2.2. Study Site, Soil Sampling, and Basic Properties

Samples (four for each soil) from two A horizons of Phaeozems were collected in August 2021: Greyic Phaeozems (Albic) under a temperate mixed forest (>70 years; dominant species were *Querqus Robur* and *Tilia Cordata*) from the V.V. Dokuchaev Soil Science Institute Ivanovsky Field Station (Russia, Tula region, 54°78′25.67″ N, 38°03′52.99″ E; additional information about the aggregate composition and mechanical properties, [35]; microbial community, [36]); and soil from the nearby arable field—four-field crop rotation by conventional tillage practice (54°45′56.1″ N, 38°01′28.9″ E) (SOM fractions, for both arable and forest soils, [37]). Since we took soil samples in August, at the end of the growing season, the harvest had already been taken and the field had not yet been ploughed. Thus, both soils were in equilibrium conditions. The mean annual precipitation is equal to 595 mm, the mean annual temperature is +6.4 °C. The parent material is cover loam. The soil textural class is Silt Loam according to USDA classification [38]. The total C contents were 39.99 ± 11.05 and 22.0 ± 0.3 g·kg^−1^, and the bulk density was 0.92 ± 0.04 and n/a g·cm^−3^, respectively. The pH_H2O_ was equal to 5.49, pH_KCl_—4.49 for the forest soil. Before analyses, samples were stored in a laboratory fridge at 4 °C for a month, then homogenized through a sieve with a mesh size of 2 mm.

### 2.3. Enzyme Activity (EA) Analyses

EA in soil suspensions was measured as hydrolytic enzyme activity of β-D-glucosidase (E.C. 3.2.1.21), cellobiosidase (E.C. 3.2.1.91), β-D-xylosidase (E.C. 3.2.1.37), and N-acetyl-β-D-glucosaminidase (E.C. 3.2.1.52) using modified fluorescent-linked substrates (4-methylumbelliferone, MUF) according to a modified Marx method [39,40]. Activity of hydrolytic enzymes were measured in black polystyrene 96-well, 300-µL microplates (Costar, Corning, New York, NY, USA). The microplates were incubated in the dark at room temperature, 24 °C, for 120 min. A CLARIOstar Plus Microplate Reader (BMG LABTECH, Ortenberg, Germany) with excitation of 360 nm and emission of 465 nm was used to determine the fluorescence of the MUF. Concentrations of the MUF were calculated using the calibration curve, and EAs were expressed as micromoles (μmol) of MUF released per g (g^−1^) of soil, per hour (h^−1^):

*EA* = (*C_MUF_ ×* vs. *× V_well_*)/(*m_ad_ × t_inc_ × V_al_*), (1)

where *C_MUF_* is the concentration of released MUF (µL·L^−1^), vs. is the volume of soil suspension (50,000 µL), *V_well_* is the total volume of liquid in each well of the microplate (200 µL), *V_al_* is the volume of aliquot of soil suspension (50 µL), *m_ad_* is the dry weight of soil (g), and *t_inc_* is time of incubation (h). The total enzyme activity was calculated for each sample as a sum of activities, determined in soil fractions with increased physical impact (Figure 1a–e).

### 2.4. Community-Level Physiological Profiling (CLPP)

Functional diversity and potential metabolic activity of microbial communities of soil suspensions separated according to the experimental scheme (Figure 1) were assessed using community-level physiological profiling (CLPP) [41,42], also known as the multisubstrate testing (MST) method [43,44].

The 96-well microplate included wells containing a set of 47 test substrates and mineral salts and one well containing distilled water with mineral salts (Table A1); the 48th well containing mineral salts without substrate was used as a control of SOM utilization [44]. Microplates were incubated in the dark in a thermostatically controlled chamber (Espec SH-241 Temperature Humidity Chamber, Osaka, Japan) at 24 °C and 99.8 air humidity for 72 h. After incubation, the optical density of the wells was measured with a CLARIOstar Plus Microplate Reader (BMG LABTECH, Ortenberg, Germany) at 450 nm wavelength.

Average well colour development (AWCD) was calculated as *AWCD* = *Σ*(*C_i_* − *R*)/47, where R is the absorbance of the control well (containing distilled water), and *C_i_* is the absorbance of the plate well inoculated with *C* source *i*. Richness was calculated as the number of oxidized substrates. The Shannon index (*H*) was calculated using an optical density value of 0.25 as the threshold for positive response: *H =* −*Σp_i_* (*lnp_i_*), where *p_i_* = (*C_i_ − R*)/*Σ*(*C_i_ − R*). The Jaccard index (*J*) was calculated as *J = c/(a + b − c)*, where *a* is the number of substrates consumed by the first microbial community, *b* is the number of substrates consumed by the second microbial community, and *c* is the number of intersected substrates consumed by the 1st and 2nd communities.

### 2.5. Statistical Analyses

Data processing and visualization were carried out in R 3.6.3. The comparison of the variables was performed with ANOVA and Tukey test in the *agricolae* package (*p* = 0.05). A significance level of *p* < 0.05 was applied. Plots were drawn in the *ggplot2* package.

## 3. Results

The total enzyme activity calculated as the sum of activities determined in all fractionated subsamples 1–5 (Figure 1) differed between land use types for *β*-glucosidase and Cellobiohydrolase, and did not differ for Chitinase or Xylanase (Table 1).

**Figure 2:**
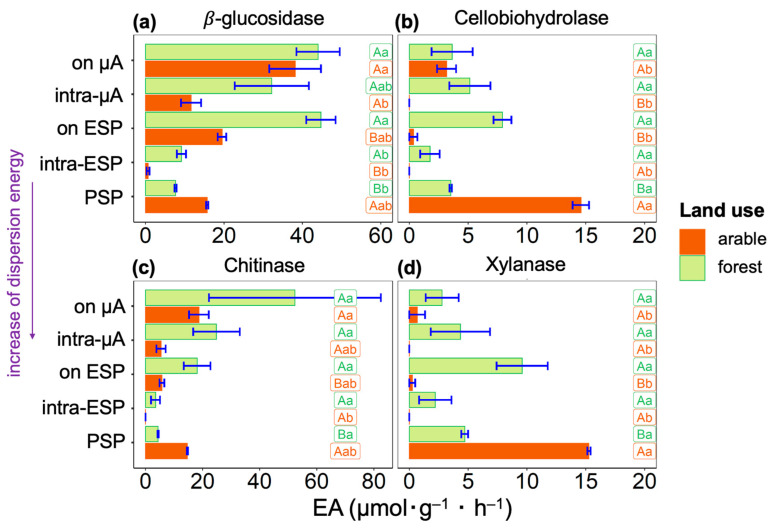
Soil enzyme activities (X axes, μM MUF (g^−1^ soil hour^−1^)) of (**a**) *β*-glucosidase, (**b**) Cellobiohydrolase, (**c**) Chitinase, and (**d**) Xylanase determined in soil suspensions, separated from A horizons of arable and forest Phaeozems with increasing dispersion energy (Y axes): on µA—from the surface of microaggregates, intra-µA—within microaggregates, on ESP—from the surface of elementary soil particles, intra-ESP—within elementary soil particles, and PSP—associated with primary soil particles. Values are mean ± SE (*n* = 4). Values followed by a different lowercase letter represent a significant difference between types of suspension. Values followed by a different capital letter represent a significant difference among land use types.

Among the four studied enzymes, the activities of *β*-glucosidase and Chitinase (Figure 2a,c) had a similar distribution within the hierarchy levels of the soil microstructure, opposite those the other two enzymes—Cellobiohydrolase and Xylanase, which were also characterized by similar distributions (Figure 2b,d). General to all four enzymes isolated from the arable soil was a low EA after high physical impact characterizing the level of elementary soil particles (intra-ESP) between the suspension isolated with minimal impact on the solids (enzymes on the microaggregates (µA) surface) and the suspension sample containing primary soil particles (PSP), obtained after maximum physical impact. For soil suspensions separated from the forest soil, no activities tended towards zero value.

The activities of *β*-glucosidase and Chitinase in both arable and forest soils were higher on the surface and within the microaggregates and elementary soil particles than their activities associated with primary soil particles (Figure 2a,c). In arable soil, for Cellobiohydrolase and Xylanase, the highest values of activities were determined in suspension with primary soil particles (Figure 2b,d). However, in the forest soil, we see the opposite for these enzymes; Cellobiohydrolase and Xylanase activities were lower in the sample with primary soil particles than in other suspensions. Therefore, in the arable soil, Cellobiohydrolase and Xylanase are associated with the surface of soil solids < 20 µm or are tightly bound to the solid surface since they are contained in a sample after being exposed to a very high (~650 J·mL^−1^) ultrasonic action. However, in the forest soil, activities of Cellobiohydrolase and Xylanase are distributed uniformly within different sites of soil microstructural units.

The activities of *β*-glucosidase and Chitinase in arable soil sample were lower in suspensions, and were associated with elementary soil particles (Figure 2a,c). In forest soil, these enzymes had the lowest activities in suspension associated with the surface of primary soil particles. The maximal activities of *β*-glucosidase and Chitinase were associated with soil microaggregates.

In forest soil, enzyme activity associated with soil solids (<20 µm) resistant to high total physical impact (~650 J·mL^−1^) was lower for *β*-glucosidase, Cellobiohydrolase, and Xylanase, and was higher for Chitinase, than the sum of activities obtained after lower physical impact (Table A2). In arable soil, enzyme activity associated with soil solids (<20 µm) resistant to high total physical impact (~650 J·mL^−1^) was lower for *β*-glucosidase and Chitinase, and was higher for Cellobiohydrolase and Xylanase, than the sum of activities obtained after lower physical impact (Table A2). Agricultural use of Phaeozem led to an increase in Cellobiohydrolase, Chitinase, and Xylanase activity tightly bound to the soil solids (<20 µm), and increased activity in Chitinase in suspensions separated with lower (<650 J·mL^−1^) ultrasonic energies.

After complete disruption of soil microaggregates to primary soil particles and the excessive impact needed to detach cells and enzymes from solids surfaces (total dispersion energy 650·mL^−1^), the total enzyme activity still associated with soil solids surface ranged from 4 to 20% for the forest soil and from 18 to 94% for the arable soil (Table A2). The proportion of activity associated with primary soil particles increased linearly for Chitinase (4%), *β*-glucosidase (6%), Cellobiohydrolase (16%), and Xylanase (20%) in forest soil, and *β*-glucosidase (18%), Chitinase (33%), Cellobiohydrolase (81%), and Xylanase (94%) in arable soil.

**Figure 3:**
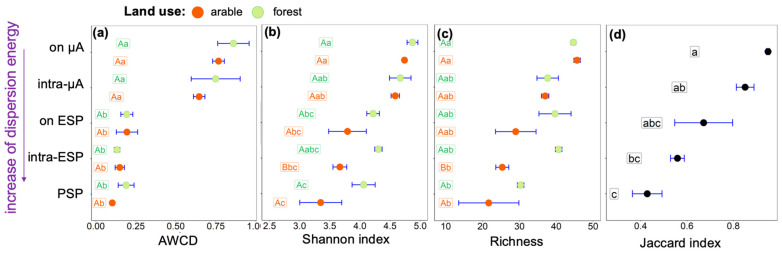
Microbial metabolic activity and diversity characterized by (**a**) Average well colour development (AWCD), (**b**) Shannon diversity index (X axis), (**c**) Richness (X axis), (**d**) Jaccard index (X axis), determined in in soil suspensions separated from A horizons of arable and forest Phaeozems with increasing dispersion energy (Y axes): on µA—from the surface of microaggregates, intra-µA—within microaggregates, on ESP—from the surface of elementary soil particles, intra-ESP—within elementary soil particles, and PSP—associated with primary soil particles. Values are mean ± SE (*n* = 4). Values followed by a different lowercase letter represent a significant difference between types of suspension. Values followed by a different capital letter represent a significant difference among land use types.

In the studied soils, potential microbial metabolic activity estimated based on AWCD was higher in soil suspensions containing cells after low mechanical impact by rotary shaker and vortex on soil solids in contrast to samples resulting from ultrasonic action (Figure 3a). We see a gradual decrease in richness with increased physical impact on soil solids (Figure 3c), and in soil samples of primary soil particles more than 20 of the 47 substrates were consumed by microorganisms. Therefore, we explain the difference in AWCD by a decrease in the microbial abundance in elementary and primary soil particles compared to soil microaggregates. With increasing physical impact, the microbial functional diversity in both forest and arable Phaeozems decreases from microaggregates to the primary soil particle level according to the Shannon index (Figure 3b) and richness (Figure 3c).

Among land use types, differences in microbial diversity were found in soil suspensions isolated after the destruction of the elementary soil particles. The lowest microbial diversity was in the arable sample, associated with intra-ESP microenvironments. The Jaccard index value increased from 0.43 in suspension with primary soil particles to 0.95 in suspension after gently shaking (Figure 3d), i.e., with increasing level of microstructural organization, the similarity of communities between land uses increases.

## 4. Discussion

Comparison of experiments using ultrasonic soil dispersion is possible when the authors specify the power of dispersion energy in W (J·s^1^). A comprehensive comparison also requires the known volume of the suspension and its concentration (soil to liquid ratio), as well as the time of ultrasound exposure, which are necessary to calculate the total energy of dispersion [45,46]. We used low-energy ultrasonic action (26 J·s^−1^) to prevent damage of microbial cells and enzymes, which is close to the physical impact used by [47,48,49]—70 J·s^−1^ power and total energy 30 J·mL^−1^—and [23,24] —50 J·s^−1^ power and total energy ~53 J·mL^−1^. Due to the gradual decrease in microbial diversity and metabolic activity with increased impact on soil samples (Figure 3b,c), we concluded that the energy of ultrasonic dispersion did not destroy microbial cells.

Soil management considerably influences the microbial activity and diversity at the microscale of the studied Phaeozems. The maximum proportion of enzyme activity in primary soil particles was found for Xylanase compared to other studied enzymes (Table A2). Moreover, for arable Phaeozem, this enzyme accounted for almost all the activity (94% of the total activity). Xylanase is a mainly extracellular enzyme associated with fungi [50], and its increased activity was previously found in coarse fractions of Cambisols, Calcic Chernozems, Luvisols [23,24], and Eutric Cambisol [51]. High activity of Xylanase in the fine fraction of arable Phaeozem could be associated with the redistribution of particulate organic matter into finer fractions. However, we suppose this to be unlikely, because (i) we used low-power ultrasonic energy; (ii) arable soil usually contains small amounts of particulate organic matter [52], whereas in the forest soil we obtained a maximum of Xylanase activity in the sample associated with the surface of elementary soil particles (Figure 2d). The increased activity of Xylanase and Cellobiohydrolase in primary soil particles of arable soil compared to those in forest soil (Table A2) might be a reflection of the substrates unavailable for decomposition, leading to enzyme accumulation on the solid surface. This is because land use results in the destruction of the soil microstructure and, consequently, an increase in the specific surface area [53].

An increased Chitinase activity at the level of microaggregates in both of the studied soils (Figure 2c) is in agreement with the predominant distribution of fungi within large, well-aerated habitats, such as the surface of microaggregates [12,50,54].

The lower the level of soil microstructure organization [5], the more specific the associated microbial community was to land use type (Figure 3d). This is opposite to the results of [47], showing the higher buffering capacity of clay fractions (<2 µm) against long-term (100 years) fertilization. In our case, the microbial diversity was reduced by agricultural activity at the level of elementary soil particles and does not differ at the microaggregate level (Figure 3b,c). At the level of primary particles, a difference was found between land use types in the activity of all four enzymes studied, and was lower in the forest soil (Figure 2).

## 5. Conclusions

We present here an experimental design based on increasing physical impact on soil solids, allowing us to characterize the microbial activity and diversity within levels of soil microstructure. The level of impact on the soil solids had a significant effect on enzyme activity and depended on the enzyme type and soil land use type. The highest proportion of activity of Xylanase and Cellobiohydrolase in arable Phaeozem was determined when dispersion energy was in the range of 450–650 J·mL^−1^, and was 94 and 81%, respectively. The highest proportions of *β*-glucosidase and Chitinase activities were determined for forest Phaeozem within the range of energies lower than 150 J·mL^−1^. Thus, the indication of soil quality based on the activity of enzymes is problematic without considering the energy of the impact on the solid phase of the soil.

Soil management considerably influenced the microbial activity and diversity at the microscale of the studied Phaeozems. The increased activity of Xylanase and Cellobiohydrolase in primary soil particles of arable soil compared to those in forest soil might be a reflection of substrates being unavailable for decomposition, leading to enzyme accumulation on the solid surface. For the Phaeozems, the lower the level of soil microstructure organization, the greater the differences between soil of different land use types, i.e., microbial communities associated with lower microstructure levels were more specific to land use type.

## Figures and Tables

**Table 1:** Total enzyme activities (μM MUF (g^−1^ soil hour^−1^)) of *β*-glucosidase, Cellobiohydrolase, Chitinase, and Xylanase determined in soil suspensions, separated from A horizons of arable and forest Phaeozems. Values are mean ± SE (*n* = 4). Values followed by a different lowercase letter show significant difference among land use types.

Enzymes Tested	Forest	Arable
*β*-glucosidase	137.70 ± 36.57 a	85.61 ± 18.80 b
Cellobiohydrolase	21.97 ± 2.17 a	18.09 ± 1.99 b
Chitinase	103.13 ± 88.49 a	44.49 ± 8.88 a
Xylanase	23.62 ± 10.85 a	16.18 ± 1.23 a

## Data Availability

The data presented in this study are available on request from the corresponding author.

## Appendix A

**Table A1:** Sole carbon sources used for CLPP.

Pentoses	Arabinose, ribose, xylose
Hexoses	Glucose, fructose, rhamnose
Oligoses	Cellobiose, lactose, maltose, sucrose
Salts of carboxylic acids	Acetate, aspartate, citrate, succinate, maleinate, pyruvate, octanoate, lactate
Amino acids	Glycine, proline, leucine, methionine, histidine, alanine, asparagine, valine, serine, phenylalanine, glutamine, arginine, lysine
Alcohols	Dulcitol, glycerol, inositol, sorbitol, mannitol
Polymers	Soluble starch, corn starch, Dextran 500, Tween 20, Tween 80, gelatin, pullulan
Miscellaneous (amides, amines phosphorylated carbons)	Creatinine, carbamide, β-glycerophosphate, glucosamine sulfate

**Table A2:** Enzyme activities (μM MUF (g^−1^ soil hour^−1^)) of *β*-glucosidase, Cellobiohydrolase, Chitinase, and Xylanase determined in samples from A horizons of arable and forest Phaeozems, and associated with particles <20 µm, separated after applying ultrasonic energy equal to 650 J·mL, and the sum of activities determined in soil suspensions obtained after applying lower energies. Values are mean ± SE (*n* = 4). Values followed by a different capital letter show significant difference among land use types. Values followed by a different lowercase letter represent significant difference between types of suspensions.

Enzymes Tested	Associated with Soil Solids < 20 µm		Sum of Activities, Determined in Supernatants	
	Forest	Arable	Forest	Arable
*β*-glucosidase	7.63 ± 0.54 Ab	15.74 ± 0.55 Bb	130.07 ± 36.48 Aa	69.87 ± 19.01 Ba
Cellobiohydrolase	3.52 ± 0.22 Bb	14.58 ± 1.39 Aa	18.46 ± 2.21 Aa	3.51 ± 0.95 Bb
Chitinase	4.39 ± 0.42 Ba	14.66 ± 0.33 Ab	98.74 ± 88.11 Aa	29.83 ± 8.82 Aa
Xylanase	4.71 ± 0.57 Bb	15.27 ± 0.28 Aa	18.92 ± 10.63 Aa	0.91 ± 1.26 Bb
